# Supplementary figures and images for: Contrasting Biogeographic Patterns of Bacterial and Archaeal Diversity in the Top- and Subsoils of Temperate Grasslands
Source: mSystems. 2019 Oct 1;4(5):e00566-19. doi: 10.1128/mSystems.00566-19 (PMC6774019; doi:10.1128/mSystems.00566-19)

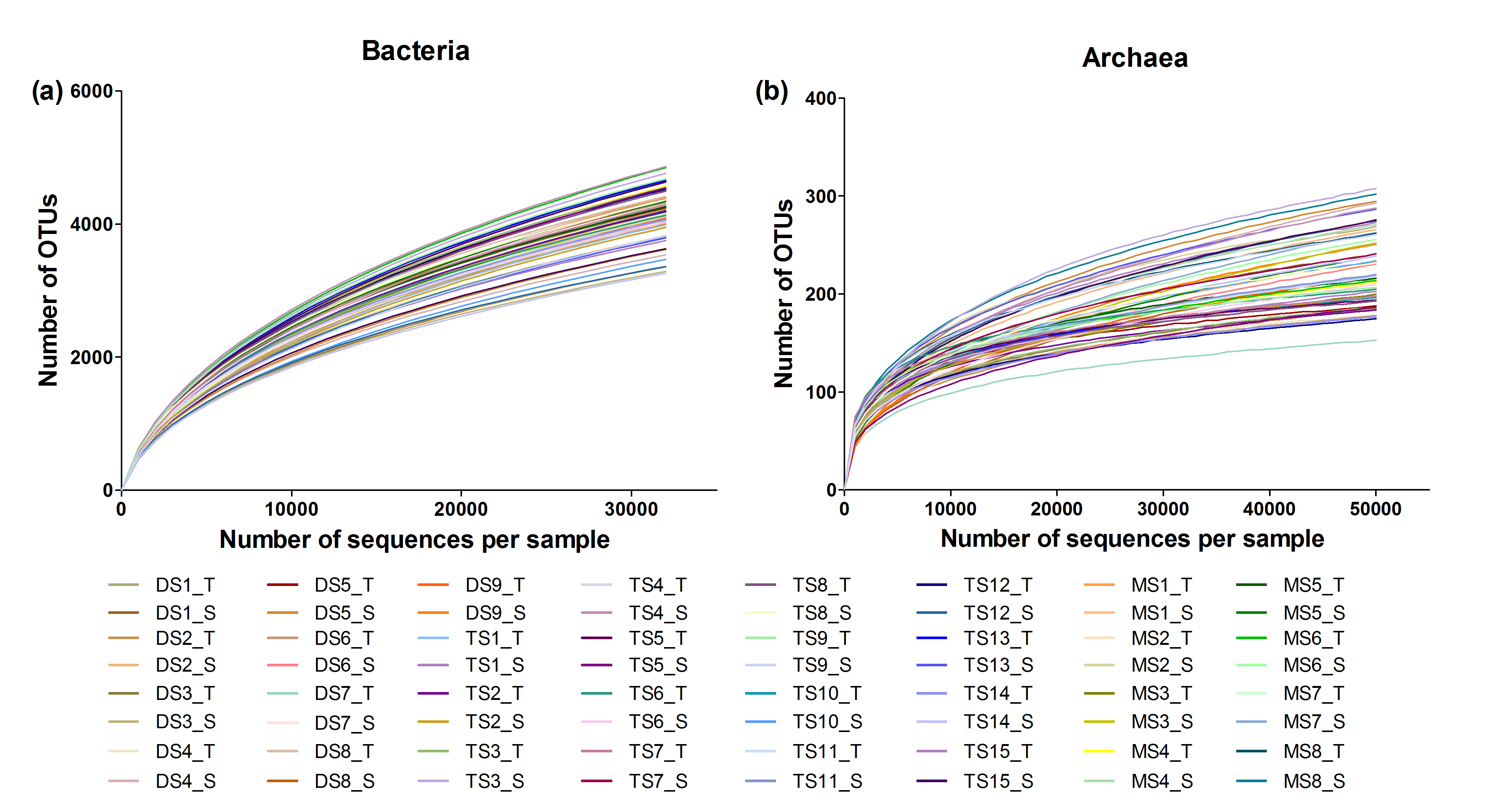

Supplement: FIG S1 [file mSystems.00566-19-sf001.tif]

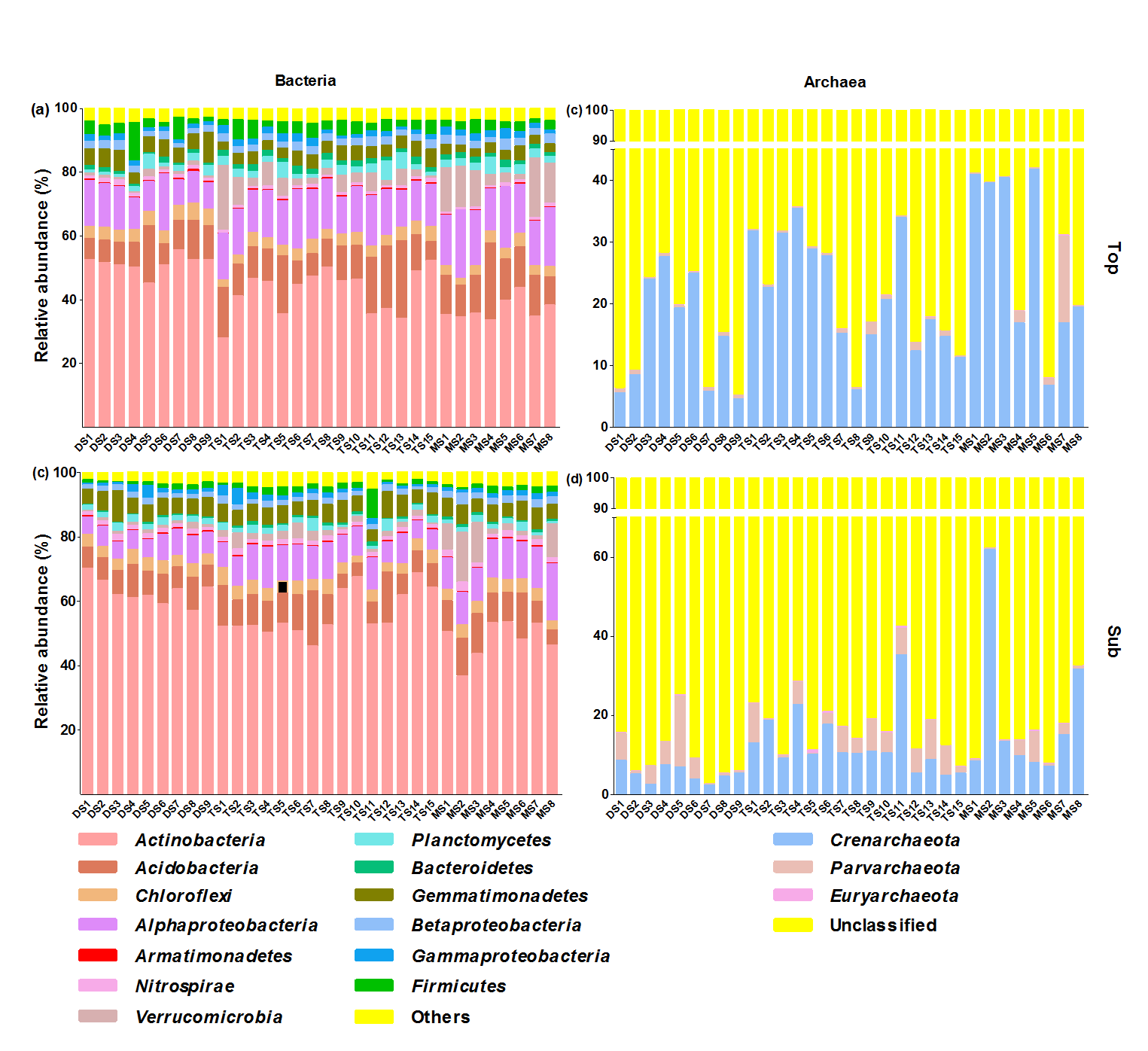

Supplement: FIG S2 [file mSystems.00566-19-sf002.tif]

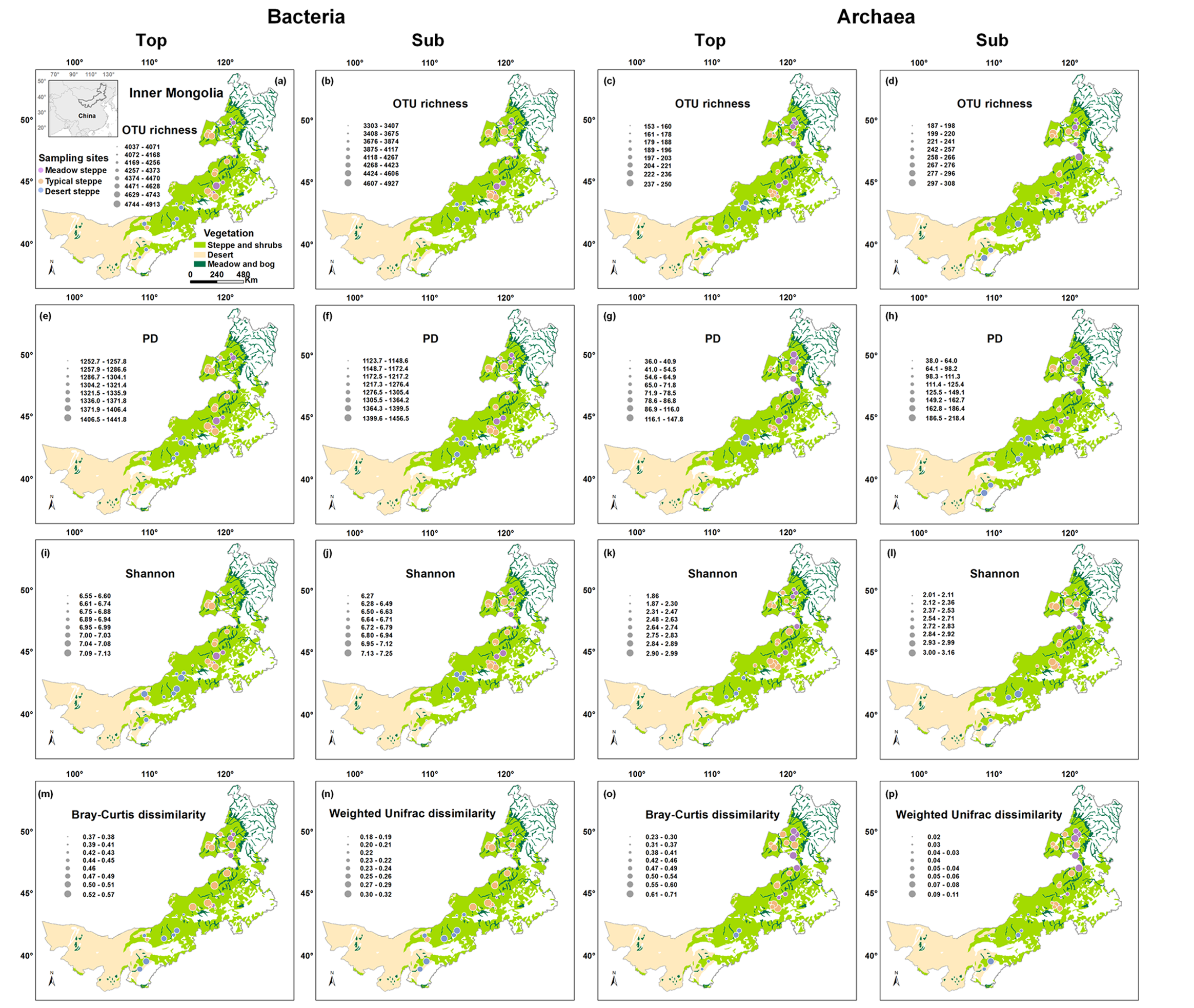

Supplement: FIG S3 [file mSystems.00566-19-sf003.tif]

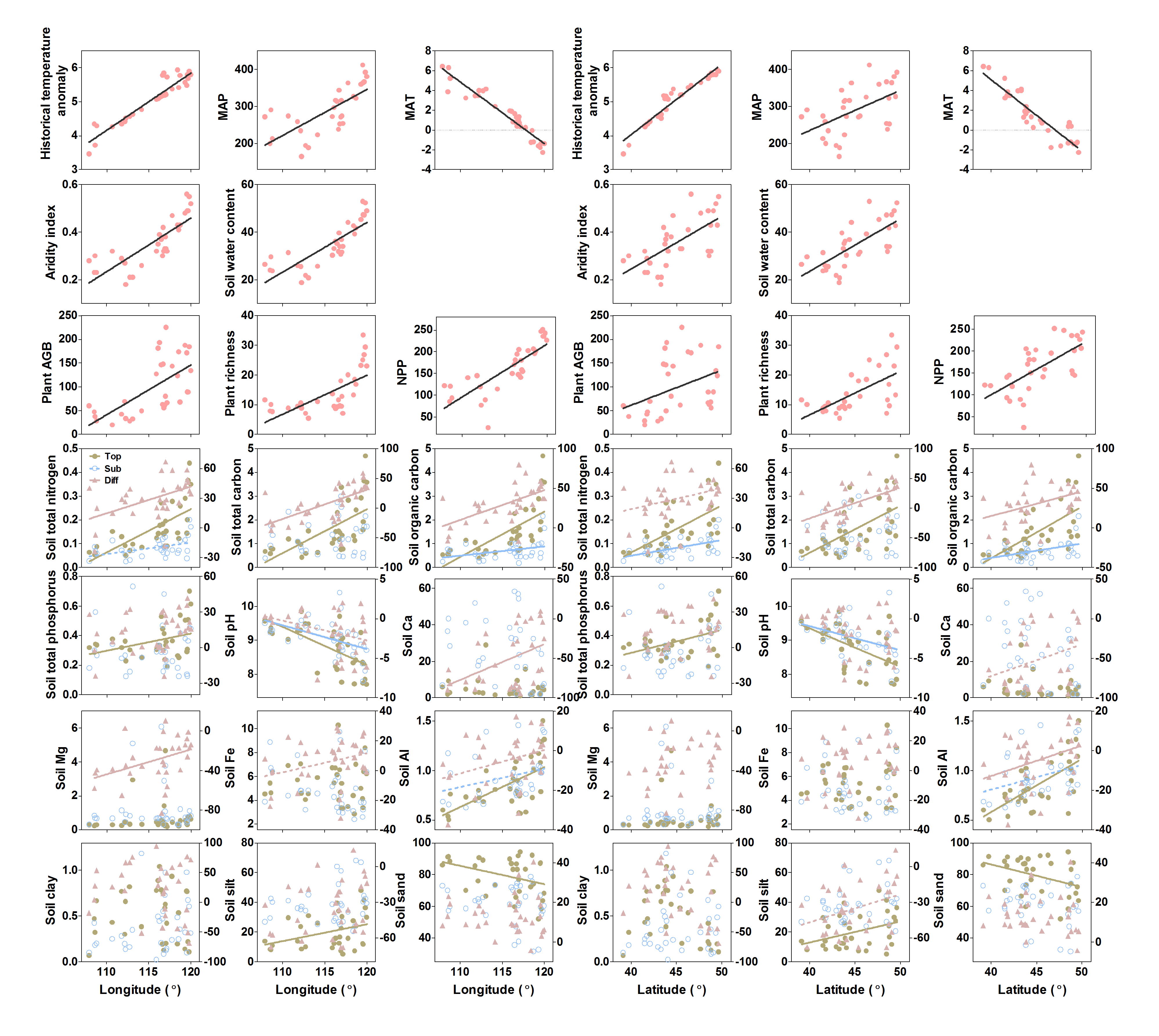

Supplement: FIG S4 [file mSystems.00566-19-sf004.tif]

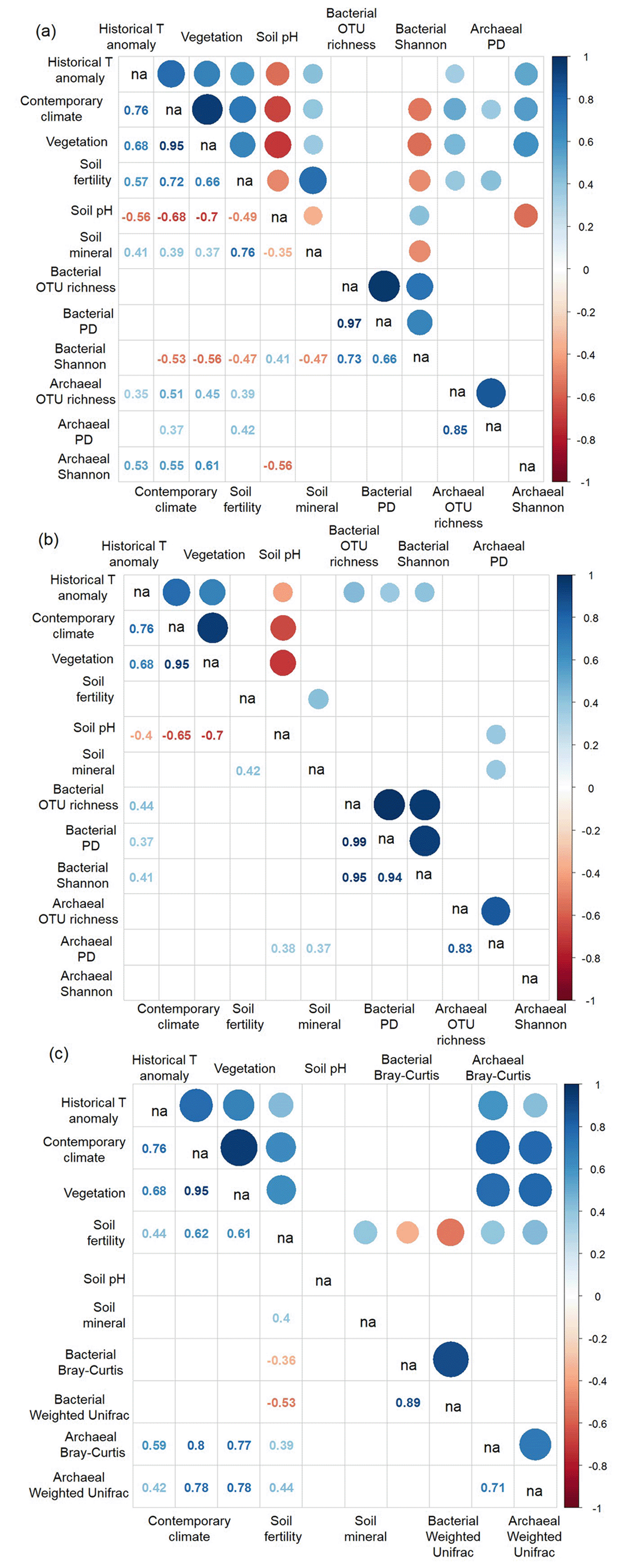

Supplement: FIG S5 [file mSystems.00566-19-sf005.tif]
