# Supplementary material for: Contrasting Biogeographic Patterns of Bacterial and Archaeal Diversity in the Top- and Subsoils of Temperate Grasslands
Source: mSystems. 2019 Oct 1;4(5):e00566-19. doi: 10.1128/mSystems.00566-19 (PMC6774019; doi:10.1128/mSystems.00566-19)
Supplement: TABLE S1 [file mSystems.00566-19-st001.docx]

**TABLE S1 Pearson correlations between soil microbial diversity and environmental variables in and between top- and subsoils.**

| Variables |  |  |  | Bacteria | | | | |  |  | |  | |  | |  | | |  | | Archaea | | | |  | |  | |  |
| --- | --- | --- | --- | --- | --- | --- | --- | --- | --- | --- | --- | --- | --- | --- | --- | --- | --- | --- | --- | --- | --- | --- | --- | --- | --- | --- | --- | --- | --- |
|  | OTU richness | | | | PD | | Shannon | | | | Community dissimilarity | | | |  | | OTU richness | | | PD | | | Shannon | | | Community dissimilarity | | | |
|  | Top | Sub | | | Top | Sub | Top | Sub | | | Bray | | Unifrac | |  | | Top | Sub | | Top | | Sub | Top | Sub | | Bray | | Unifrac | |
| T anomaly | 0.18 | **0.44*** | | | 0.24 | **0.37*** | -0.28 | **0.41*** | | | -0.03 | | -0.22 | |  | | **0.35*** | -0.08 | | 0.24 | | -0.09 | **0.53**** | -0.07 | | **0.59***** | | **0.42*** | |
| MAP | -0.10 | 0.25 | | | -0.10 | 0.20 | **-0.51**** | 0.11 | | | -0.07 | | -0.29 | |  | | **0.50**** | 0.05 | | 0.33 | | -0.02 | **0.48**** | 0.11 | | **0.80***** | | **0.79***** | |
| MAT | 0.00 | **-0.37*** | | | -0.05 | -0.29 | **0.48**** | -0.31 | | | 0.03 | | 0.26 | |  | | **-0.41*** | 0.05 | | -0.31 | | 0.10 | **-0.53**** | 0.03 | | **-0.70***** | | **-0.61***** | |
| AI | -0.12 | 0.27 | | | -0.11 | 0.21 | **-0.53**** | 0.22 | | | -0.09 | | -0.32 | |  | | **0.52**** | 0.04 | | **0.38*** | | -0.02 | **0.53**** | 0.01 | | **0.80***** | | **0.79***** | |
| SWC | -0.10 | 0.27 | | | -0.09 | 0.20 | **-0.53**** | 0.21 | | | -0.09 | | -0.31 | |  | | **0.52**** | 0.04 | | **0.39*** | | -0.01 | **0.51**** | -0.01 | | **0.78***** | | **0.79***** | |
| Plant SR | -0.30 | 0.04 | | | -0.26 | -0.02 | **-0.72***** | 0.03 | | | 0.10 | | -0.19 | |  | | **0.39*** | 0.22 | | **0.43*** | | 0.25 | 0.28 | -0.14 | | **0.57***** | | **0.78***** | |
| Plant AGB | 0.11 | **0.44*** | | | 0.08 | **0.38*** | -0.22 | **0.44*** | | | -0.16 | | -0.28 | |  | | 0.35 | -0.25 | | 0.16 | | -0.35 | **0.64***** | 0.15 | | **0.60***** | | **0.47**** | |
| Plant NPP | -0.03 | 0.32 | | | -0.04 | 0.25 | **-0.50**** | 0.27 | | | -0.03 | | -0.28 | |  | | **0.44*** | 0.02 | | 0.24 | | -0.09 | **0.66***** | 0.14 | | **0.81***** | | **0.77***** | |
| Soil TN | -0.16 | 0.02 | | | -0.12 | -0.01 | **-0.50**** | 0.04 | | | **-0.39*** | | **-0.52**** | |  | | **0.38*** | 0.07 | | 0.34 | | 0.00 | **0.35*** | 0.12 | | 0.25 | | 0.31 | |
| Soil TC | -0.10 | 0.05 | | | -0.06 | 0.05 | **-0.43*** | 0.02 | | | -0.19 | | -0.34 | |  | | **0.41*** | 0.27 | | **0.46**** | | 0.25 | 0.13 | 0.12 | | **0.62***** | | **0.61***** | |
| Soil OC | -0.19 | -0.03 | | | -0.15 | -0.06 | **-0.54**** | -0.02 | | | -0.34 | | **-0.50**** | |  | | **0.38*** | 0.08 | | **0.37*** | | 0.04 | 0.32 | 0.02 | | **0.39*** | | **0.37*** | |
| Soil TP | -0.23 | 0.06 | | | -0.17 | 0.08 | -0.29 | 0.04 | | | **-0.35*** | | **-0.51**** | |  | | 0.28 | 0.31 | | **0.41*** | | **0.41*** | -0.15 | -0.14 | | 0.13 | | 0.27 | |
| Soil pH | 0.06 | 0.02 | | | 0.04 | 0.06 | **0.41*** | 0.10 | | | -0.16 | | -0.13 | |  | | -0.32 | 0.31 | | -0.19 | | **0.38*** | **-0.56***** | -0.02 | | -0.01 | | 0.08 | |
| Soil Ca | 0.25 | -0.13 | | | 0.25 | -0.11 | 0.24 | -0.15 | | | -0.31 | | -0.34 | |  | | 0.17 | **0.36*** | | **0.36*** | | 0.25 | **-0.45**** | 0.34 | | 0.32 | | **0.40*** | |
| Soil Mg | 0.25 | **0.36*** | | | 0.29 | **0.37*** | 0.18 | 0.31 | | | -0.10 | | -0.16 | |  | | 0.20 | 0.22 | | **0.42*** | | **0.41*** | **-0.45*** | -0.31 | | 0.31 | | 0.31 | |
| Soil Fe | -0.08 | -0.21 | | | -0.09 | -0.17 | -0.06 | -0.22 | | | -0.04 | | -0.12 | |  | | -0.16 | **0.41*** | | -0.17 | | 0.34 | 0.10 | -0.03 | | 0.08 | | 0.16 | |
| Soil Al | -0.27 | -0.11 | | | -0.17 | -0.12 | **-0.59***** | -0.10 | | | -0.02 | | -0.14 | |  | | 0.31 | 0.23 | | **0.38*** | | 0.28 | 0.20 | -0.17 | | 0.14 | | 0.21 | |
| Soil clay | -0.14 | -0.13 | | | -0.04 | -0.14 | 0.07 | -0.16 | | | -0.16 | | -0.10 | |  | | 0.28 | -0.03 | | 0.31 | | -0.11 | -0.15 | -0.01 | | -0.20 | | -0.20 | |
| Soil silt | -0.23 | 0.29 | | | -0.20 | 0.27 | **-0.46**** | 0.25 | | | -0.01 | | -0.07 | |  | | 0.19 | 0.34 | | 0.28 | | **0.37*** | 0.01 | 0.06 | | 0.20 | | 0.06 | |
| Soil sand | 0.24 | -0.28 | | | 0.20 | -0.27 | **0.45**** | -0.24 | | | -0.20 | | -0.21 | |  | | -0.21 | -0.34 | | -0.29 | | **-0.37*** | -0.00 | -0.06 | | -0.13 | | -0.02 | |
| The one, two and three asterisks mean significant correlation at *p* < 0.05, *p* < 0.01, and *p* < 0.001. Bray, Bray-Curtis dissimilarity in microbial community between top- and subsoil; Unifrac, Weighted Unifrac dissimilarity in microbial community between top- and subsoil; T anomaly, historical temperature anomaly; MAP, mean annual precipitation; MAT, mean annual temperature; AI, aridity index, calculated as AI = MAP / PET; PET, potential evapotranspiration; SWC, soil water content (mm month^-1^); plant SR, plant species richness; plant AGB, plant aboveground biomass; plant NPP, plant net primary productivity; soil TN, soil total nitrogen; soil TC, soil total carbon; soil OC, soil organic carbon; soil TP, soil total phosphorus; soil Ca, soil calcium; soil Mg, soil magnesium; soil Fe, soil iron; soil Al, soil aluminum. | | | | | | | | | | | | | | | | | | | | | | | | | | | | |  |
