# Supplementary material for: Contrasting Biogeographic Patterns of Bacterial and Archaeal Diversity in the Top- and Subsoils of Temperate Grasslands
Source: mSystems. 2019 Oct 1;4(5):e00566-19. doi: 10.1128/mSystems.00566-19 (PMC6774019; doi:10.1128/mSystems.00566-19)
Supplement: TABLE S2 [file mSystems.00566-19-st002.docx]

**TABLE S2 First principal component (PC 1) extracted from the principal component analysis (**PCA**) for the five environmental groups and results of Kaise-Meyer-Olkin (KMO) test and Bartlett test of sphericity (BS) for variables used for principle component analysis (PCA) in the paper..** The plus (+) and minus (-) in the parentheses refer to the positive and negative correlation between the PC1 and the individual variables.

| Pincomp | Parameters | PC 1 (%) | | | KMO values | | | BS test | | |
| --- | --- | --- | --- | --- | --- | --- | --- | --- | --- | --- |
| Contemporary climate PC 1 | MAP (+), MAT (-), Aridity index (+), SWC (+) | 92.6 | | | 0.68 | | | *χ*^2^ = 292, *p* < 0.05 | | |
| Vegetation PC 1 | Plant species richness (+), aboveground biomass (+), NPP (+) | 75.3 | | | 0.57 | | | *χ*^2^ = 45, *p* < 0.05 | | |
|  | | **Top** | **Sub** | **Diss** | **Top** | **Sub** | **Diss** | **Top** | **Sub** | **Diss** |
| Soil fertility PC 1 of topsoil | Soil total carbon (+), soil organic carbon (+), soil total nitrogen (+), soil total phosphorus (+) | 88.7 | 68.7 | 79.9 | 0.82 | 0.66 | 0.72 | *χ*^2^ = 206,  *p* < 0.05 | *χ*^2^ = 97,  *p* < 0.05 | *χ*^2^ = 120,  *p* < 0.05 |
| Soil mineral PC 1 | Soil silt(+), soil sand(-), soil extractable Fe (+), soil extractable Al (+) | 74.5 |  | 62.2 | 0.69 |  | 0.63 | *χ*^2^ = 209, *p* < 0.05 |  | *χ*^2^ = 53,  *p* < 0.05 |
|  | Soil silt(+), soil sand(-), soil extractable Fe (+) |  | 66.7% |  |  | 0.50 |  |  | *χ*^2^ = 221, *p* < 0.05 |  |

MAP, mean annual precipitation; MAT, mean annual temperature; SWC, soil water content; NPP, net primary productivity; Fe, iron; Al, aluminum; Top, topsoil; Sub, subsoil; Diss, top-subsoil community dissimilarity.
