# Supplementary material for: Contrasting Biogeographic Patterns of Bacterial and Archaeal Diversity in the Top- and Subsoils of Temperate Grasslands
Source: mSystems. 2019 Oct 1;4(5):e00566-19. doi: 10.1128/mSystems.00566-19 (PMC6774019; doi:10.1128/mSystems.00566-19)
Supplement: TEXT S1 [file mSystems.00566-19-s0001.docx]

**Supplementary Information**

**Contrasting biogeographic patterns of bacterial and archaeal diversity in the top- and subsoils of temperate grasslands**

**Running title: Microbial diversity patterns in top- and subsoils**

Nana Liu^a,b^, Huifeng Hu^b^, Wenhong Ma^c^, Ye Deng^d^, Yuqing Liu^b^, Baihui Hao^c^, Xinying Zhang^b^, Dimitar Dimitrov^e^, Xiaojuan Feng^b,*^, Zhiheng Wang^a,*^

^a^ Institute of Ecology and Key Laboratory for Earth Surface Processes of the Ministry of Education, College of Urban and Environmental Sciences, Peking University, Beijing 100871, China

^b^ State Key Laboratory of Vegetation and Environmental Change, Institute of Botany, Chinese Academy of Sciences, Beijing 100093, China

^c^ College of Ecology and Environment, Inner Mongolia University, Hohhot, 010021, China

^d^ CAS Key Laboratory of Environmental Biotechnology, Research Center for Eco-Environmental Sciences, Chinese Academy of Sciences, Beijing 100085, China

^e^ Department of Natural History, University Museum of Bergen, University of Bergen, Bergen, Norway

*Authors for correspondence: Zhiheng Wang ([zhiheng.wang@pku.edu.cn](mailto:zhiheng.wang@pku.edu.cn)); Xiaojuan Feng ([xfeng@ibcas.ac.cn](mailto:xfeng@ibcas.ac.cn))

**Changes in climate and soil properties along the transect**

Most climate and vegetation variables, such as historical T anomaly since LGM (Last Glacial Maximum), MAP (Mean Annual Precipitation), AI (aridity index), SWC (soil water content), plant species richness (SR), aboveground biomass (AGB) and ecosystem net primary productivity (NPP) significantly increased from southwest towards northeast along this transect (Fig. S4, all *p* < 0.05). MAT (Mean Annual Temperature) significantly decreased from southwest towards northeast in the same geographic direction (*p* < 0.05). Soil fertility-related properties all increased in topsoil (i.e. total nitrogen, TN; total carbon, TC; total phosphorus, TP; and partial organic carbon, OC; Fig. S4, all *p* < 0.05) and subsoil (TN, 0.1 < *p* < 0.05; OC, *p* < 0.05) from southwest towards northeast. They all (except soil TC) had higher concentrations in the topsoil in most sites, and their concentrations differed between pairwise top- and subsoils, with increasing (*p* < 0.05) with both longitude and latitude (except TN with latitude, *p* > 0.1) from 0 towards larger positive values. It suggested that the concentrations differenced in these variables between pairwise top- and subsoils were larger at eastern regions. Compared with other soil fertility-related variables, the relative difference in TC between top- and subsoils increased with longitude from ca. -50 to ca. 50, suggesting that TC had higher concentration in topsoil than subsoil in southwest sites, but the opposite was true in the northeast sites (Fig. S4). Soil pH decreased both in top- and subsoil from southwest towards northeast, and the subsoil had higher pH than the topsoil at most sites. However, the difference in pH between pairwise top- and subsoil decreased with longitude (0.05 < *p* < 0.1) (Fig. S4). Soil Ca and Mg showed little geographic variation both in top- and subsoil from southwest towards northeast. In most sites, the subsoil had higher concentrations of available Ca and Mg than the topsoil, and their concentration differences between pairwise top- and sub-soil significantly increased with longitude (*p* < 0.05), and only soil available Ca marginally significantly increased with latitude (0.05 < *p* < 0.1) towards zero in the east (Fig. S4). Soil Fe and Al showed little and increased changes in top- and subsoils from southwest towards northeast. There was more soil available Fe in the topsoil than subsoil at most sites and the difference marginally significantly increased with longitude (0.05 < *p* < 0.1) but not with latitude. In the western sites, the subsoil had higher soil Al (top < sub), whereas in the eastern sites the topsoil had higher Al concentration (top > sub). Thus, the difference of soil Al significantly increased (*p* < 0.05) with latitude and marginally significantly (0.05 < *p* < 0.1) increased with longitude. Soil silt and sand significantly increased and decreased only in topsoil from southwest towards northeast. Topsoil had less silt, but more sand than subsoil, and the difference between topsoil and subsoil silt marginally significantly increased with latitude (Fig. S4).
